# Supplementary material for: A Likelihood Approach for Real-Time Calibration of Stochastic Compartmental Epidemic Models
Source: PLoS Comput Biol. 2017 Jan 17;13(1):e1005257. doi: 10.1371/journal.pcbi.1005257 (PMC5240920; doi:10.1371/journal.pcbi.1005257)
Supplement: S1 File — (TAR.GZ) [file pcbi.1005257.s014.tar.gz › HSPH_Online-SI-Revision/Additions/MSS11/n100-extreme-SEITR_noisy/n100-extreme-SEITR_noisy_NEW_table4.pdf]

1	R0	{1.38654, 9.85565, 0.1}	{2.93978, 19.9817, 0.}	{{2.93978, 19.9817, 0.}, {2.93978, 19.9817, 0.}}	{112.022, 102.744, 100.}	inf	4758.	13 434.9	{13 338.4, 13 531.8}	182.364	0.450803	newly	2726.36	4171.51	{4081., 4244.}	53.0064	0.184665	Reff	0.128602	0.317073	{0.315442, 0.318475}	146.555	ar	92 379.	93 605.1	{93 513., 93 687.}	1.32725
2	R0	{2.36629, 12.2029, 0.1}	{2.70795, 10.355, 0.}	{{2.70795, 10.355, 0.}, {2.70795, 10.355, 0.}}	{14.4388, 15.1435, 100.}	inf	5892.	5345.82	{5276.35, 5431.64}	9.2699	0.042275	newly	2664.	2845.04	{2775., 2903.}	6.7958	0.0284748	Reff	0.0301229	0.274455	{0.273223, 0.275756}	811.118	ar	99 022.	91 648.3	{91 567., 91 730.}	7.44649
3	R0	{2.32253, 12.836, 1.}	{2.91946, 11.8546, 2.}	{{2.91946, 11.8546, 2.}, {2.91946, 11.8546, 2.}}	{25.7015, 7.64553, 100.}	inf	7824.	7905.53	{7795.54, 7992.74}	1.16907	0.00503727	newly	3413.	3764.88	{3702., 3833.}	10.31	0.0425671	Reff	0.035767	0.256534	{0.255187, 0.257902}	617.237	ar	98 997.	93 466.	{93 374., 93 546.}	5.587
4	R0	{1.79075, 11.2058, 1.}	{2.74056, 10.6768, 0.}	{{2.74056, 10.6768, 0.}, {2.74056, 10.6768, 0.}}	{53.0397, 4.72117, 100.}	inf	6713.	6073.33	{5991.84, 6138.69}	9.52882	0.0435121	newly	3304.	3156.7	{3097., 3217.}	4.45823	0.0198534	Reff	0.0739401	0.275234	{0.273852, 0.276483}	272.24	ar	96 920.	91 953.7	{91 885., 92 038.}	5.12411
5	R0	{2.26952, 9.62371, 0.1}	{2.77394, 8.24535, 0.}	{{2.77394, 8.24535, 0.}, {2.77394, 8.24535, 0.}}	{22.2258, 14.3225, 100.}	inf	2480.	2234.05	{2184.44, 2284.08}	9.91739	0.0454279	newly	1318.	1390.99	{1335., 1439.}	5.58498	0.0234697	Reff	0.0289591	0.232456	{0.231673, 0.233214}	702.704	ar	98 881.	92 266.9	{92 213., 92 314.}	6.68894
6	R0	{1.57191, 6.89004, 0.1}	{2.99092, 10.3743, 0.}	{{2.99092, 10.3743, 0.}, {2.99092, 10.3743, 0.}}	{90.2727, 50.5697, 100.}	inf	1451.	4240.83	{4168.87, 4307.73}	192.269	0.465745	newly	1057.	2214.92	{2156., 2269.}	109.548	0.3212	Reff	0.0825567	0.210706	{0.209333, 0.211716}	155.225	ar	95 046.	93 977.8	{93 914., 94 046.}	1.12383
7	R0	{1.99226, 17.2339, 1.}	{2.88523, 18.2127, 0.}	{{2.88523, 18.2127, 0.}, {2.88523, 18.2127, 0.}}	{44.8219, 5.67928, 100.}	inf	11 476.	11 413.1	{11 315.8, 11 520.1}	0.717504	0.00313059	newly	3977.	3835.79	{3753., 3913.}	3.55117	0.0157552	Reff	0.0686533	0.303194	{0.301662, 0.30447}	341.63	ar	97 992.	93 201.3	{93 093., 93 289.}	4.88883
8	R0	{2.63369, 15.1879, 0.1}	{2.80387, 10.6536, 0.}	{{2.80387, 10.6536, 0.}, {2.80387, 10.6536, 0.}}	{6.46153, 29.8546, 100.}	inf	8203.	5026.07	{4934.12, 5108.59}	38.7289	0.212777	newly	3068.	2601.88	{2526., 2668.}	15.193	0.0716446	Reff	0.022123	0.251379	{0.250108, 0.252669}	1036.28	ar	99 451.	92 533.	{92 445., 92 622.}	6.95621
9	R0	{2.53271, 9.90552, 0.1}	{2.77394, 8.24535, 0.}	{{2.77394, 8.24535, 0.}, {2.77394, 8.24535, 0.}}	{9.52454, 16.76, 100.}	inf	2578.	2229.26	{2180.02, 2271.34}	13.5274	0.0631781	newly	1330.	1387.85	{1339., 1431.}	4.43233	0.0187229	Reff	0.0191979	0.232534	{0.231586, 0.233599}	1111.25	ar	99 355.	92 258.4	{92 193., 92 319.}	7.14264
10	R0	{2.48791, 18.9003, 2.}	{2.86001, 13.5542, 0.}	{{2.86001, 13.5542, 0.}, {2.86001, 13.5542, 0.}}	{14.9562, 28.2856, 100.}	inf	14 986.	9621.51	{9534.7, 9708.1}	35.7966	0.192454	newly	4707.	4139.53	{4065., 4209.}	12.0559	0.0558343	Reff	0.0381148	0.287266	{0.285049, 0.288942}	653.686	ar	99 291.	93 010.6	{92 931., 93 094.}	6.32529
11	R0	{2.84414, 18.4765, 1.}	{2.52588, 12.2207, 0.}	{{2.52588, 12.2207, 0.}, {2.52588, 12.2207, 0.}}	{11.1899, 33.858, 100.}	inf	13 352.	8159.88	{8066.64, 8254.43}	38.8865	0.21388	newly	4255.	3898.09	{3818., 3966.}	8.38801	0.0380976	Reff	0.0214164	0.353489	{0.351977, 0.355055}	1550.55	ar	99 654.	89 602.6	{89 463., 89 712.}	10.0863
12	R0	{2.29369, 14.622, 0.1}	{2.73634, 13.3345, 1.}	{{2.73634, 13.3345, 1.}, {2.73634, 13.3345, 1.}}	{19.2987, 8.80542, 900.}	inf	7894.	8094.55	{8031.69, 8163.86}	2.54057	0.0108851	newly	3139.	3547.	{3475., 3620.}	12.9978	0.0530151	Reff	0.0347723	0.298136	{0.296843, 0.299299}	757.395	ar	98 982.	91 915.9	{91 813., 92 008.}	7.13875
13	R0	{2.73413, 16.7529, 1.}	{2.9884, 12.8326, 0.}	{{2.9884, 12.8326, 0.}, {2.9884, 12.8326, 0.}}	{9.29971, 23.4007, 100.}	inf	10 283.	7452.21	{7373.61, 7516.56}	27.5288	0.139848	newly	3594.	3316.62	{3245., 3379.}	7.71786	0.0349323	Reff	0.0212989	0.238955	{0.237499, 0.24044}	1021.92	ar	99 543.	93 968.4	{93 881., 94 037.}	5.60021
14	R0	{1.79554, 16.5943, 2.}	{2.56757, 17.005, 0.}	{{2.56757, 17.005, 0.}, {2.56757, 17.005, 0.}}	{42.9973, 2.47477, 100.}	inf	13 533.	12 300.3	{12 193.7, 12 403.3}	9.10877	0.0414868	newly	4850.	4475.62	{4390., 4548.}	7.71918	0.0349369	Reff	0.106063	0.404975	{0.403212, 0.406691}	281.827	ar	96 821.	90 093.4	{89 962., 90 209.}	6.94846
15	R0	{1.91429, 13.7771, 1.}	{2.81891, 18.2812, 0.}	{{2.81891, 18.2812, 0.}, {2.81891, 18.2812, 0.}}	{47.256, 32.693, 100.}	inf	8092.	13 402.2	{13 287.1, 13 495.1}	65.6228	0.219113	newly	3380.	4529.54	{4444., 4586.}	34.0101	0.127105	Reff	0.0667896	0.347333	{0.345557, 0.349123}	420.04	ar	97 541.	92 661.5	{92 563., 92 774.}	5.00254
16	R0	{1.42094, 10.5871, 2.}	{2.93371, 17.1903, 1.}	{{2.93371, 17.1903, 1.}, {2.93371, 17.1903, 1.}}	{106.462, 62.3702, 50.}	inf	7426.	12 812.2	{12 720.9, 12 918.3}	72.5318	0.236861	newly	4015.	4528.84	{4443., 4609.}	12.798	0.0522667	Reff	0.134932	0.309928	{0.30839, 0.311545}	129.691	ar	93 044.	93 580.9	{93 475., 93 664.}	0.57705
17	R0	{1.45515, 6.16466, 0.1}	{2.17292, 6.16453, 0.}	{{2.17292, 6.16453, 0.}, {2.17292, 6.16453, 0.}}	{49.3258, 0.0020887, 100.}	inf	1735.	1965.01	{1909.72, 2024.39}	13.2571	0.0539649	newly	1376.	1597.14	{1543., 1649.}	16.0712	0.0645903	Reff	0.105091	0.376167	{0.374834, 0.377395}	257.944	ar	93 254.	83 806.9	{83 684., 83 908.}	10.1305
18	R0	{2.85669, 13.2416, 0.1}	{2.9194, 10.1535, 0.}	{{2.9194, 10.1535, 0.}, {2.9194, 10.1535, 0.}}	{2.19533, 23.3211, 100.}	inf	6133.	4328.49	{4264.68, 4384.77}	29.423	0.151364	newly	2583.	2305.06	{2255., 2361.}	10.7604	0.0495139	Reff	0.014512	0.223505	{0.222631, 0.224321}	1440.14	ar	99 644.	93 466.	{93 404., 93 530.}	6.20008
19	R0	{2.56417, 17.9558, 0.1}	{2.7499, 15.3124, 0.}	{{2.7499, 15.3124, 0.}, {2.7499, 15.3124, 0.}}	{7.24317, 14.7216, 100.}	inf	13 822.	13 088.1	{12 978., 13 202.}	5.30986	0.023703	newly	4524.	5169.26	{5074., 5254.}	14.263	0.0578714	Reff	0.0335137	0.36202	{0.360313, 0.363737}	980.215	ar	99 389.	92 037.7	{91 920., 92 170.}	7.39648
20	R0	{2.27517, 11.1864, 0.1}	{2.99092, 10.3743, 0.}	{{2.99092, 10.3743, 0.}, {2.99092, 10.3743, 0.}}	{31.459, 7.25957, 100.}	inf	4328.	4244.49	{4173.93, 4306.55}	1.97764	0.00870215	newly	2013.	2215.77	{2156., 2268.}	10.073	0.0415998	Reff	0.0313746	0.210476	{0.209366, 0.211649}	570.85	ar	98 894.	93 985.1	{93 919., 94 063.}	4.9638
21	R0	{2.35735, 10.4735, 0.1}	{2.77394, 8.24535, 0.}	{{2.77394, 8.24535, 0.}, {2.77394, 8.24535, 0.}}	{17.6719, 21.2741, 100.}	inf	3720.	2238.59	{2177.37, 2290.04}	39.8228	0.220642	newly	1848.	1397.45	{1353., 1442.}	24.3804	0.121473	Reff	0.0275103	0.232396	{0.231487, 0.233166}	744.76	ar	99 036.	92 264.7	{92 210., 92 320.}	6.83721
22	R0	{1.79551, 11.9179, 2.}	{2.73634, 13.3345, 1.}	{{2.73634, 13.3345, 1.}, {2.73634, 13.3345, 1.}}	{52.3991, 11.8861, 50.}	inf	6227.	8090.57	{8017.27, 8173.05}	29.9272	0.113686	newly	2882.	3545.57	{3481., 3600.}	23.0246	0.0899494	Reff	0.0710663	0.298071	{0.296423, 0.299503}	319.427	ar	97 033.	91 910.3	{91 824., 91 999.}	5.27934
23	R0	{1.23545, 6.31687, 0.1}	{2.26582, 8.12562, 0.}	{{2.26582, 8.12562, 0.}, {2.26582, 8.12562, 0.}}	{83.4007, 28.6336, 100.}	inf	1659.	2292.27	{2238.8, 2348.61}	38.1717	0.140343	newly	1370.	1509.15	{1458., 1558.}	10.1569	0.0418666	Reff	0.148353	0.352087	{0.351089, 0.353159}	137.331	ar	88 739.	85 628.8	{85 548., 85 719.}	3.50494
24	R0	{2.24089, 10.1789, 0.1}	{2.93906, 10.3908, 0.}	{{2.93906, 10.3908, 0.}, {2.93906, 10.3908, 0.}}	{31.1558, 2.08158, 100.}	inf	4192.	4879.87	{4797.41, 4954.79}	16.4091	0.0659515	newly	2182.	2550.12	{2491., 2600.}	16.8708	0.0676321	Reff	0.0325825	0.224741	{0.223422, 0.225996}	589.76	ar	98 868.	93 616.6	{93 545., 93 689.}	5.31148
25	R0	{1.57298, 10.2607, 0.1}	{2.52588, 12.2207, 0.}	{{2.52588, 12.2207, 0.}, {2.52588, 12.2207, 0.}}	{60.5796, 19.1023, 100.}	inf	6559.	8146.1	{8056.09, 8243.92}	24.1973	0.0940939	newly	3489.	3889.49	{3812., 3968.}	11.4786	0.0471449	Reff	0.10572	0.35345	{0.351839, 0.355119}	234.326	ar	94 957.	89 601.3	{89 458., 89 715.}	5.64012
26	R0	{1.2154, 8.2576, 0.1}	{2.7499, 15.3124, 0.}	{{2.7499, 15.3124, 0.}, {2.7499, 15.3124, 0.}}	{126.255, 85.4342, 100.}	inf	3875.	9660.23	{9571.18, 9739.3}	149.296	0.396705	newly	2648.	3779.27	{3709., 3850.}	42.7217	0.154446	Reff	0.165416	0.31386	{0.312539, 0.315045}	89.7399	ar	88 421.	92 032.1	{91 921., 92 145.}	4.08398
27	R0	{2.22841, 18.7983, 2.}	{2.7499, 15.3124, 0.}	{{2.7499, 15.3124, 0.}, {2.7499, 15.3124, 0.}}	{23.4018, 18.5436, 100.}	inf	12 941.	9670.64	{9582.83, 9751.71}	25.2713	0.126523	newly	4125.	3778.68	{3707., 3850.}	8.39564	0.038134	Reff	0.0518997	0.31389	{0.312521, 0.315297}	504.801	ar	98 756.	92 031.3	{91 946., 92 122.}	6.80946
28	R0	{1.27778, 5.36767, 1.}	{2.68628, 9.02342, 1.}	{{2.68628, 9.02342, 1.}, {2.68628, 9.02342, 1.}}	{110.23, 68.1068, 0.}	inf	1113.	3350.85	{3287.83, 3419.53}	201.065	0.478602	newly	965.	1971.08	{1918., 2030.}	104.257	0.310069	Reff	0.136275	0.259878	{0.25879, 0.261032}	90.7006	ar	89 843.	91 409.8	{91 336., 91 488.}	1.74398
29	R0	{2.38042, 15.8901, 2.}	{2.71225, 12.7047, 1.}	{{2.71225, 12.7047, 1.}, {2.71225, 12.7047, 1.}}	{13.9399, 20.0464, 50.}	inf	11 729.	9123.01	{9022.33, 9212.79}	22.2184	0.109135	newly	4278.	4171.3	{4079., 4263.}	2.57317	0.0113665	Reff	0.0399673	0.316066	{0.314409, 0.317712}	690.813	ar	99 112.	91 684.5	{91 576., 91 803.}	7.49401
30	R0	{2.53188, 12.7607, 0.1}	{2.99092, 10.3743, 0.}	{{2.99092, 10.3743, 0.}, {2.99092, 10.3743, 0.}}	{18.1302, 18.701, 100.}	inf	8098.	7170.65	{7080.13, 7252.59}	11.4516	0.0528369	newly	3519.	3768.29	{3691., 3849.}	7.08411	0.0296684	Reff	0.0262556	0.235745	{0.234165, 0.237367}	797.885	ar	99 317.	93 987.5	{93 918., 94 047.}	5.36615
31	R0	{1.24482, 10.5471, 1.}	{2.52268, 16.0877, 0.}	{{2.48143, 15.7026, 0.}, {2.48143, 15.7026, 0.}}	{102.654, 52.5321, 100.}	inf	5996.	9970.78	{9549.12, 9761.34}	66.2905	0.218707	newly	3288.	3776.9	{3671., 3852.}	14.8692	0.0599397	Reff	0.174511	0.382083	{0.385381, 0.389947}	118.944	ar	89 097.	89 422.3	{88 874., 89 189.}	0.569795
32	R0	{2.30446, 17.9607, 1.}	{2.72475, 13.1605, 0.87}	{{2.64721, 11.9965, 0.}, {2.73634, 13.3345, 1.}}	{18.2383, 26.7259, 13.}	inf	13 243.	7997.14	{7461.89, 8158.77}	39.6123	0.219246	newly	4308.	3544.6	{3477., 3615.}	17.7205	0.0847578	Reff	0.0453748	0.299885	{0.296759, 0.310645}	560.907	ar	99 028.	91 790.9	{91 001., 92 007.}	7.30817
33	R0	{2.31579, 11.8236, 2.}	{2.77394, 8.24535, 0.}	{{2.77394, 8.24535, 0.}, {2.77394, 8.24535, 0.}}	{19.7837, 30.2636, 100.}	inf	4389.	2236.96	{2179.89, 2280.01}	49.0326	0.292769	newly	2012.	1391.57	{1346., 1437.}	30.8365	0.16026	Reff	0.0280905	0.232295	{0.231361, 0.233147}	726.952	ar	99 012.	92 268.	{92 210., 92 332.}	6.81131
34	R0	{1.6678, 10.6744, 1.}	{2.71225, 12.7047, 1.}	{{2.71225, 12.7047, 1.}, {2.71225, 12.7047, 1.}}	{62.6243, 19.0204, 0.}	inf	4684.	6197.68	{6113.8, 6268.2}	32.3161	0.121594	newly	2446.	2816.29	{2754., 2877.}	15.1386	0.0611604	Reff	0.0850078	0.282456	{0.281055, 0.283827}	232.271	ar	95 809.	91 686.1	{91 592., 91 772.}	4.30321
35	R0	{1.65476, 7.07559, 0.1}	{2.77394, 8.24535, 0.}	{{2.77394, 8.24535, 0.}, {2.77394, 8.24535, 0.}}	{67.6339, 16.5324, 100.}	inf	1687.	2228.9	{2163.86, 2279.41}	32.1222	0.120907	newly	1151.	1390.5	{1338., 1446.}	20.808	0.0819597	Reff	0.0754074	0.23233	{0.231435, 0.233206}	208.1	ar	95 746.	92 270.1	{92 203., 92 315.}	3.63037
36	R0	{2.71807, 18.5959, 0.1}	{2.53075, 11.6597, 0.}	{{2.53075, 11.6597, 0.}, {2.53075, 11.6597, 0.}}	{6.89168, 37.2995, 100.}	inf	15 586.	8591.15	{8513.53, 8682.06}	44.8791	0.258695	newly	5029.	4265.73	{4192., 4343.}	15.1774	0.071529	Reff	0.0291921	0.357998	{0.356528, 0.359656}	1126.35	ar	99 540.	89 668.2	{89 560., 89 786.}	9.91738
37	R0	{2.6202, 12.7552, 0.1}	{2.9884, 12.8326, 0.}	{{2.9884, 12.8326, 0.}, {2.9884, 12.8326, 0.}}	{14.0522, 0.606786, 100.}	inf	6696.	7453.78	{7369.43, 7527.44}	11.3169	0.0465468	newly	2840.	3319.86	{3255., 3375.}	16.8965	0.0677505	Reff	0.0220097	0.238988	{0.237692, 0.240632}	985.83	ar	99 426.	93 969.	{93 892., 94 046.}	5.48853
38	R0	{1.47672, 9.81651, 1.}	{2.52368, 12.5037, 0.}	{{2.52368, 12.5037, 0.}, {2.52368, 12.5037, 0.}}	{70.8975, 27.3743, 100.}	inf	4663.	6626.27	{6546.39, 6708.89}	42.1031	0.152583	newly	2643.	3088.66	{3017., 3153.}	16.8619	0.0676027	Reff	0.109425	0.3338	{0.332455, 0.33503}	205.049	ar	93 815.	89 573.7	{89 479., 89 675.}	4.5209
39	R0	{1.90004, 12.3313, 1.}	{2.64721, 11.9965, 0.}	{{2.64721, 11.9965, 0.}, {2.64721, 11.9965, 0.}}	{39.3241, 2.71487, 100.}	inf	7298.	7420.47	{7335.2, 7505.}	1.69871	0.00730184	newly	3317.	3558.16	{3494., 3637.}	7.27043	0.0304382	Reff	0.0681354	0.311738	{0.310063, 0.313258}	357.527	ar	97 413.	91 002.1	{90 904., 91 095.}	6.58117
40	R0	{2.64218, 18.5237, 2.}	{2.99939, 13.7076, 1.}	{{2.99939, 13.7076, 1.}, {2.99939, 13.7076, 1.}}	{13.5196, 25.9995, 50.}	inf	11 629.	7540.11	{7439.26, 7615.07}	35.1611	0.188182	newly	3674.	3181.55	{3125., 3247.}	13.4036	0.0625544	Reff	0.0267917	0.237445	{0.236224, 0.238652}	786.265	ar	99 474.	94 052.3	{93 965., 94 129.}	5.45041
41	R0	{1.6945, 7.17996, 0.1}	{2.17292, 6.16453, 0.}	{{2.17292, 6.16453, 0.}, {2.17292, 6.16453, 0.}}	{28.2334, 14.1425, 100.}	inf	2490.	1971.09	{1911.25, 2030.96}	20.84	0.101614	newly	1635.	1598.12	{1541., 1654.}	2.96269	0.0131269	Reff	0.0694067	0.376041	{0.374769, 0.377165}	441.794	ar	96 332.	83 817.4	{83 695., 83 932.}	12.9911
42	R0	{1.84559, 18.0074, 1.}	{2.81891, 18.2812, 0.}	{{2.81891, 18.2812, 0.}, {2.81891, 18.2812, 0.}}	{52.7374, 1.52076, 100.}	inf	13 679.	13 389.9	{13 309.2, 13 495.8}	2.11363	0.00928423	newly	4650.	4517.79	{4444., 4600.}	2.84323	0.0125659	Reff	0.093313	0.347325	{0.345531, 0.348763}	272.215	ar	97 358.	92 668.5	{92 564., 92 764.}	4.81681
43	R0	{2.24965, 17.0361, 2.}	{2.86001, 13.5542, 0.}	{{2.86001, 13.5542, 0.}, {2.86001, 13.5542, 0.}}	{27.1312, 20.4382, 100.}	inf	13 450.	9598.51	{9501.82, 9683.08}	28.6356	0.14653	newly	4598.	4139.24	{4064., 4208.}	9.97738	0.0456886	Reff	0.0537891	0.287275	{0.285665, 0.288832}	434.076	ar	98 770.	92 993.4	{92 881., 93 084.}	5.84851
44	R0	{2.54324, 15.7967, 0.1}	{2.64721, 11.9965, 0.}	{{2.64721, 11.9965, 0.}, {2.64721, 11.9965, 0.}}	{4.0882, 24.0568, 100.}	inf	8875.	7428.52	{7345.02, 7512.31}	16.2984	0.0772841	newly	3246.	3556.66	{3470., 3632.}	9.57055	0.0396352	Reff	0.0255087	0.311837	{0.31041, 0.313359}	1122.47	ar	99 354.	90 998.2	{90 886., 91 087.}	8.41009
45	R0	{2.54943, 14.8032, 0.1}	{2.80387, 10.6536, 0.}	{{2.80387, 10.6536, 0.}, {2.80387, 10.6536, 0.}}	{9.98014, 28.0317, 100.}	inf	8157.	5018.96	{4946.15, 5110.8}	38.4706	0.210948	newly	3187.	2600.07	{2541., 2663.}	18.4164	0.0884595	Reff	0.0250609	0.251385	{0.249954, 0.252666}	903.095	ar	99 380.	92 526.	{92 446., 92 613.}	6.89679
46	R0	{2.37755, 12.6461, 2.}	{2.17292, 6.16453, 0.}	{{2.17292, 6.16453, 0.}, {2.17292, 6.16453, 0.}}	{8.60697, 51.2535, 100.}	inf	7240.	1966.19	{1910.77, 2033.76}	72.8426	0.566253	newly	3124.	1595.94	{1540., 1651.}	48.9136	0.291887	Reff	0.0309795	0.376155	{0.374727, 0.377367}	1114.21	ar	99 096.	83 812.	{83 697., 83 937.}	15.4235
47	R0	{1.69279, 11.6636, 1.}	{2.52588, 12.2207, 0.}	{{2.52588, 12.2207, 0.}, {2.52588, 12.2207, 0.}}	{49.2143, 4.77662, 100.}	inf	7476.	8150.05	{8054.95, 8247.77}	9.01613	0.0374719	newly	3657.	3896.95	{3821., 3966.}	6.56139	0.0275529	Reff	0.0899041	0.353252	{0.351639, 0.354745}	292.921	ar	96 214.	89 594.9	{89 473., 89 710.}	6.87953
48	R0	{1.82402, 10.0038, 1.}	{2.74056, 10.6768, 0.}	{{2.74056, 10.6768, 0.}, {2.74056, 10.6768, 0.}}	{50.2483, 6.727, 100.}	inf	5228.	6065.51	{6004.35, 6122.87}	16.0196	0.0645159	newly	2763.	3153.79	{3095., 3210.}	14.1437	0.0574044	Reff	0.0676164	0.275109	{0.273732, 0.276538}	306.867	ar	97 076.	91 954.	{91 865., 92 037.}	5.27626
49	R0	{1.6424, 11.1667, 2.}	{2.71225, 12.7047, 1.}	{{2.71225, 12.7047, 1.}, {2.71225, 12.7047, 1.}}	{65.1393, 13.7732, 50.}	inf	4808.	6186.99	{6104.35, 6268.7}	28.6811	0.109493	newly	2454.	2810.94	{2735., 2871.}	14.5452	0.0589041	Reff	0.0891166	0.282509	{0.280996, 0.283636}	217.01	ar	95 546.	91 678.9	{91 607., 91 749.}	4.04734
50	R0	{2.05638, 14.3048, 1.}	{2.9884, 12.8326, 0.}	{{2.9884, 12.8326, 0.}, {2.9884, 12.8326, 0.}}	{45.3232, 10.2917, 100.}	inf	9256.	7449.85	{7370.29, 7525.62}	19.5133	0.0942887	newly	3752.	3318.04	{3247., 3386.}	11.5661	0.0534311	Reff	0.0564271	0.238666	{0.237322, 0.240171}	322.964	ar	98 194.	93 969.3	{93 895., 94 036.}	4.30237
